# Efficacy and safety of hepatic arterial infusion chemotherapy combined with tyrosine kinase inhibitors and PD-1 inhibitors for unresectable hepatocellular carcinoma: a meta-analysis

**DOI:** 10.3389/fonc.2026.1896692

**Published:** 2026-10-08

**Authors:** Renbin Ouyang, Xiaocheng Li, Kun Huang, Duke Chen, Yaowei Yang, Xinmin Li, Liming Zhu, Chunzhi Liang

**Affiliations:** 1 Department of Hepatobiliary Surgery, Loudi Central Hospital, Loudi, China; 2 Department of Hepatobiliary Surgery, The First Affiliated Hospital of Hunan University of Medicine, Huaihua, China; 3 Department of Hepatobiliary Surgery, The Second Affiliated Hospital of Chongqing Medical University, Chongqing, China; 4 Department of General Surgery, The People’s Hospital of Liangping District, Chongqing, China; 5 Department of Gynecologic Oncology, Chongqing University Cancer Hospital, Chongqing, China; 6 Department of General Surgery, University-Town Hospital of Chongqing Medical University, Chongqing, China; 7 Department of Gastroenterology and Hepatology, Shanghai Ninth People’s Hospital, Shanghai Jiao Tong University School of Medicine, Shanghai, China; 8 Department of Hematology and Oncology, The First Affiliated Hospital of Hunan University of Medicine, Huaihua, China

**Keywords:** hepatic arterial infusion chemotherapy, hepatocellular carcinoma, meta-analysis, programmed cell death-1, tyrosine kinase inhibitor

## Abstract

**Background:**

The triplet combination of hepatic arterial infusion chemotherapy (HAIC), a tyrosine kinase inhibitor (TKI), and a programmed cell death-1 (PD-1) inhibitor has emerged as a highly promising strategy for unresectable hepatocellular carcinoma (uHCC). This study aimed to comprehensively evaluate the efficacy and safety of this triple-therapy compared with the dual-therapy of a TKI plus a PD-1 inhibitor.

**Methods:**

We systematically searched PubMed, Embase, and the Cochrane Library for randomized controlled trials or comparative cohort studies that assessed HAIC plus a TKI and a PD-1 inhibitor (H-T-P) versus a TKI plus a PD-1 inhibitor (T-P) for uHCC. The primary endpoints were overall survival (OS), progression-free survival (PFS), objective response rate (ORR), and disease control rate (DCR). The secondary endpoint was treatment-related adverse events (AEs). A random-effects model was employed to pool data, calculating hazard ratios (HRs) or relative risks (RRs) with their 95% confidence intervals (CIs).

**Results:**

A total of 12 studies involving 2,386 patients with uHCC were included. Compared with T-P dual therapy, the H-T-P triple therapy was associated with significantly improved OS (HR, 0.51; 95%CI, 0.47-0.56; *P* < 0.001) and PFS (HR, 0.59; 95%CI, 0.51-0.68; *P* < 0.001). Furthermore, the H-T-P group demonstrated a significantly higher ORR (RR, 1.97; 95%CI, 1.73-2.24; *P* < 0.001) and DCR (RR, 1.19; 95%CI, 1.11-1.28; *P* < 0.001). Regarding safety, while no significant difference was observed in the overall incidence of all-grade AEs (*P* = 0.238), the incidence of grade ≥3 AEs was significantly higher in the triple-therapy arm (RR, 1.29; 95%CI, 1.13-1.48; *P* = 0.004), consisting primarily of manageable hepatotoxicity and hematologic toxicities.

**Conclusion:**

Our findings indicate that for patients with uHCC, the H-T-P triple-therapy regimen is superior to dual therapy in terms of survival benefits and tumor response. The associated increase in severe toxicity is considered predictable and clinically manageable.

**Systematic Review Registration:**

https://www.crd.york.ac.uk/PROSPERO/, identifier CRD420251113276.

## Introduction

Hepatocellular carcinoma (HCC) represents a major global health burden and ranks among the leading causes of cancer-related mortality worldwide (1). A substantial proportion of patients are diagnosed at an intermediate to advanced stage, where curative interventions such as surgical resection, ablation, or liver transplantation are no longer feasible (2). For this population of patients with unresectable hepatocellular carcinoma (uHCC), systemic therapy constitutes the cornerstone of management (1). Over the past decade, the landscape of systemic treatment for uHCC has undergone significant evolution (3). Oral multi-target tyrosine kinase inhibitors (TKIs), exemplified by sorafenib and lenvatinib, were once the established first-line standard of care (4, 5). More recently, advances in the understanding of the tumor immune microenvironment have propelled immune checkpoint inhibitors (ICIs), particularly those targeting programmed cell death-1 (PD-1) and its ligand (PD-L1), to the forefront of uHCC treatment (6, 7). The pivotal IMbrave150 trial established the combination of atezolizumab (an anti-PD-L1 antibody) with bevacizumab (an anti-VEGF antibody) as a new first-line standard, demonstrating superior overall survival (OS) compared with sorafenib (8, 9). However, despite these remarkable advances, outcomes for high-risk subpopulations—such as those with main trunk portal vein tumor thrombosis (Vp4 PVTT)—remain suboptimal. Recent updated data from the IMbrave150 trial reported a median OS of only 7.6 months for patients with Vp4 PVTT receiving atezolizumab plus bevacizumab (10). This highlights a critical therapeutic gap for patients with a massive tumor burden and severe vascular invasion. Similarly, the HIMALAYA study showed that dual immunotherapy with durvalumab (anti-PD-L1) and tremelimumab (anti-CTLA-4) also conferred a survival advantage over sorafenib (11). Building on these breakthroughs, regimens combining a TKI with a PD-1 inhibitor have shown considerable potential in numerous clinical trials and real-world studies, leading to their widespread adoption in clinical practice owing to their synergistic effects (12).

Concurrently, hepatic arterial infusion chemotherapy (HAIC) has garnered considerable attention as a potent locoregional therapy. This approach enables the delivery of high-concentration chemotherapeutic agents directly to hepatic tumors, thereby minimizing systemic toxicity (13). Particularly in Asian populations, HAIC with FOLFOX-based regimens has demonstrated efficacy in patients with advanced HCC (13, 14). Mechanistically, HAIC is theorized to induce immunogenic cell death, leading to the release of tumor-associated antigens and subsequently amplifying the host’s anti-tumor immune response (15). This local immunomodulatory effect may act in synergy with systemic PD-1 inhibition (16). Furthermore, TKIs can contribute by inhibiting angiogenesis and favorably modulating the tumor microenvironment to facilitate the infiltration and activation of immune cells (15).

Based on this compelling rationale, a triple-therapy regimen (H-T-P) comprising HAIC, a TKI, and a PD-1 inhibitor has emerged. This strategy aims to achieve synergistic anti-cancer activity through a multi-faceted approach involving potent local tumor debulking, anti-angiogenesis, and systemic immune activation (15). In recent years, a number of retrospective studies and small-scale clinical trials have reported encouraging efficacy and a manageable safety profile for this triplet combination (15, 17, 18). However, these studies have generally been limited by small sample sizes and predominantly single-arm or non-randomized comparative designs. Consequently, whether this approach is superior to the current mainstream dual-therapy regimen of a TKI plus a PD-1 inhibitor (T-P) is not yet supported by high-level evidence.

Therefore, we conducted this systematic review and meta-analysis to quantitatively synthesize all available evidence. Our objective was to compare the efficacy and safety of the H-T-P triple-therapy versus the T-P dual-therapy in patients with uHCC, with the goal of providing a reliable evidence base to inform clinical decision-making and guide future research.

## Materials and methods

This meta-analysis was conducted and reported in accordance with the Preferred Reporting Items for Systematic Reviews and Meta-Analyses (PRISMA) statement (19). The protocol for this review was prospectively registered with the International Prospective Register of Systematic Reviews (PROSPERO; Registration No. CRD420251113276).

### Search strategy

A comprehensive literature search was performed across the PubMed, Embase, and Cochrane Library databases for relevant studies published from database inception to July 2025. The search strategy was constructed using a combination of Medical Subject Headings (MeSH) and free-text terms centered on the key concepts of HCC, HAIC, TKIs, and ICIs. Core search terms included “hepatocellular carcinoma”, “HCC”, “hepatic arterial infusion chemotherapy”, “HAIC”, “tyrosine kinase inhibitor”, “TKI”, “programmed cell death-1”, “PD-1”, and “immune checkpoint inhibitor”. This strategy was adapted to the specific syntax of each database. Furthermore, the reference lists of all included articles and relevant systematic reviews were manually scrutinized to identify additional eligible studies. For the manual reference screening workflow, two reviewers independently evaluated the reference lists. The same pre-defined inclusion and exclusion criteria were rigorously applied: initially screening by title and abstract, followed by full-text retrieval and detailed assessment for ultimate eligibility. The detailed search strategy for each database is provided in **Supplementary Table 1**.

### Inclusion and exclusion criteria

Two reviewers (CL and XL) independently screened the titles and abstracts of the retrieved literature, followed by a full-text assessment of potentially eligible studies. Any discrepancies were resolved by consensus or through consultation with a third, senior reviewer (YY).

Studies were included if they met the following criteria: (1) involved patients with uHCC confirmed by histopathological or radiological evidence; (2) the experimental group received triple therapy with HAIC, a TKI, and a PD-1 inhibitor; (3) the control group received dual therapy with a TKI and a PD-1 inhibitor; (4) reported at least one of the following outcomes: OS, progression-free survival (PFS), objective response rate (ORR), disease control rate (DCR), or treatment-related adverse events (AEs); and (5) were either randomized controlled trials (RCTs) or comparative cohort studies (prospective or retrospective).

The exclusion criteria were: (1) reviews, case reports, letters, editorials, and commentaries; (2) studies with inappropriate interventions or comparators; (3) studies from which necessary data could not be reliably extracted; and (4) duplicate publications, in which case the most comprehensive or recent report was retained.

### Data extraction and quality assessment

Data extraction was performed independently by two reviewers using a pre-specified, standardized electronic form. The extracted information included: (1) study characteristics: first author, year of publication, country/region, and study design; (2) baseline patient characteristics: sample size, age, sex, Child-Pugh class, Barcelona Clinic Liver Cancer (BCLC) stage, and the presence of extrahepatic metastasis; and (3) outcome data: hazard ratios (HRs) with 95% confidence intervals (CIs) for OS and PFS; the number of events and total patients for ORR and DCR in each group; and the number of specific AEs per group.

The methodological quality of the included studies was independently assessed by the same two reviewers. The Cochrane Risk of Bias 2 (RoB 2) tool was used for RCTs (20). For non-randomized cohort studies, the Newcastle-Ottawa Scale (NOS) was employed, wherein a score of ≥7 (out of a maximum of 9) was considered to indicate a low risk of bias (21).

### Statistical analysis

All statistical analyses were conducted using the meta and metafor packages in R software (version 4.4.0). HRs were used to pool time-to-event data (OS and PFS), while risk ratios (RRs) were used for dichotomous outcomes (ORR, DCR, and AEs). All pooled effect estimates are presented with their corresponding 95% CIs. Statistical heterogeneity among studies was evaluated using the Cochran’s Q test and the *I*^2^ statistic (22). A *P*-value < 0.05 for the Q test was considered to indicate significant heterogeneity, and the *I*^2^ statistic was used to quantify its magnitude. Given the anticipated clinical and methodological diversity across studies, a random-effects model was applied for all analyses. To provide more conservative estimates, particularly when the number of studies was limited, the Hartung-Knapp-Sidik-Jonkman (HKSJ) adjustment was applied (23, 24).

Potential publication bias was assessed by visual inspection of funnel plots and formally tested using Egger’s regression test (25) and Harbord’s regression test (26, 27). Where significant bias was detected, the trim-and-fill method was employed as a sensitivity analysis to estimate and adjust for the potential impact of missing studies (28). Furthermore, a leave-one-out sensitivity analysis was performed to evaluate the influence of each individual study on the overall pooled estimate. For all primary analyses, a two-tailed *P*-value < 0.05 was considered statistically significant.

## Results

### Study selection

The initial systematic search yielded 551 records. After the removal of 156 duplicates, 333 articles were excluded based on title and abstract screening. This left 62 full-text articles for eligibility assessment. Following full-text review, 50 studies were excluded for the following reasons: ineligible intervention or comparator (n=22), inappropriate study design (n=14), unavailable or insufficient outcome data (n=11), or redundant publication (n=3). Ultimately, 12 studies were included in the quantitative synthesis (29–40). The study selection process is depicted in the PRISMA flow diagram (**Figure 1**).

**Figure 1:**
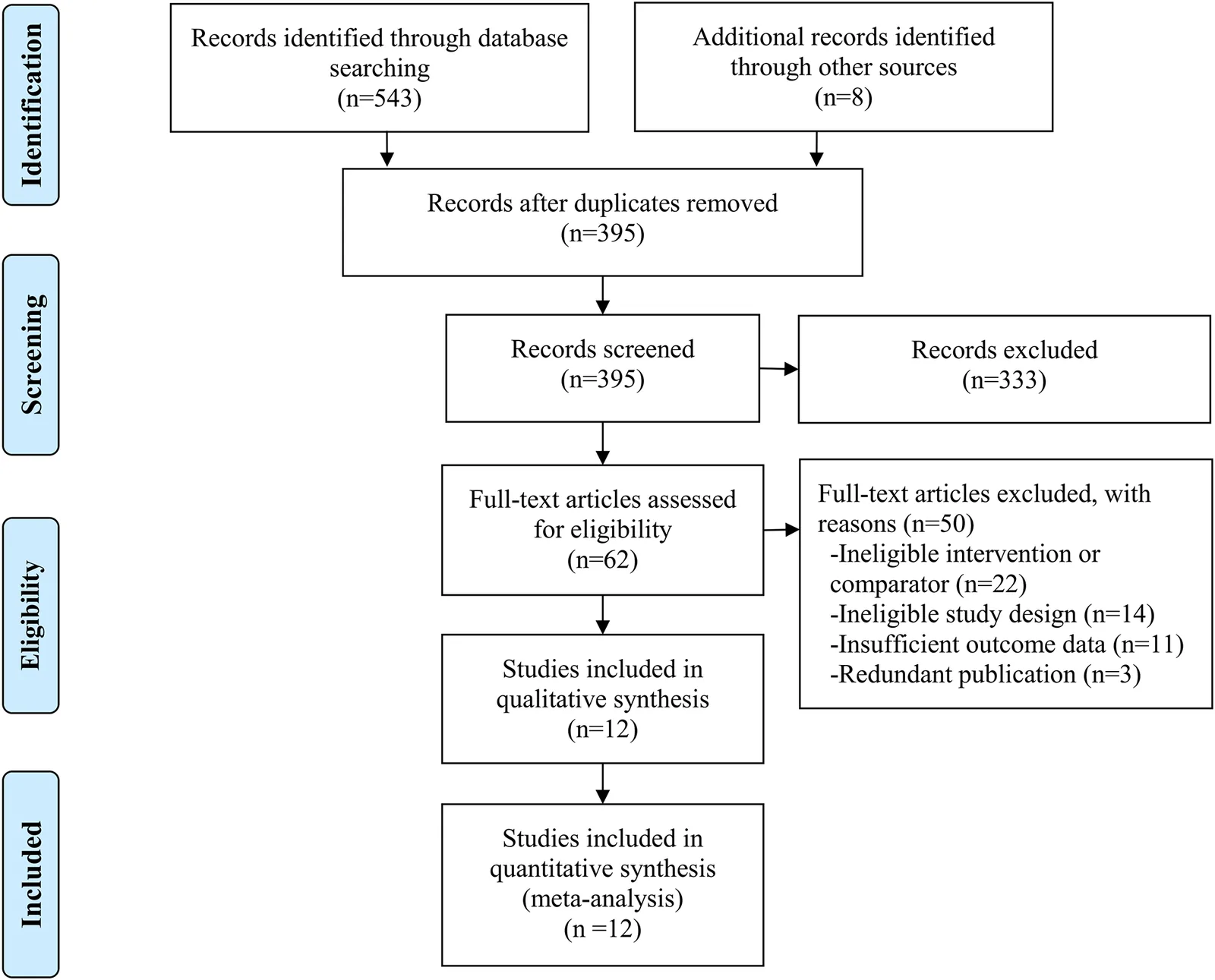
Flowchart of study selection.

### Characteristics of included studies

This meta-analysis incorporated 12 studies published between 2021 and 2025, encompassing a total of 2,386 patients with a confirmed diagnosis of advanced or unresectable HCC. Of these patients, 1,275 received H-T-P therapy and 1,111 received T-P therapy. Among the included studies, one was a prospective cohort study, while the remainder were retrospective in design. One publication, a conference abstract, provided data only for OS, PFS, and ORR, with information on DCR and AEs being unavailable; complete data were extracted from all other studies. The HAIC regimen was FOLFOX-based in all included studies. The TKIs administered included lenvatinib, sorafenib, apatinib, and donafenib, with lenvatinib being the most frequently used. The PD-1 inhibitors included camrelizumab, sintilimab, tislelizumab, toripalimab, and pembrolizumab. Detailed baseline characteristics of the studies are summarized in **Table 1**. The quality of the included studies was assessed using the NOS; each study achieved a score of ≥7, indicating a low risk of bias (**Supplementary Table 2**).

**Table 1:** Baseline characteristics of the studies.

Study ID	Study design	Treatment regimen	No. of patients	Age (mean ± SD, year)	Male/female	BCLC stage (B/C)	Child-Pugh class (A/B)	Extrahepatic metastasis (no/yes)
Zuo M (2025)	RCS	HAIC+TKI+PD-1	245	≤50/>50:133/112	220/25	C:245	222/23	111/134
		TKI+PD-1	221	≤50/>50:116/105	182/39	C:221	199/22	123/98
Lou Y (2025)	RCS	HAIC+Len+PD-1	67	56.15 ± 7.78	61/6	C:67	56/11	27/40
		Len+PD-1	48	53.48 ± 10.39	44/4	C:48	37/11	13/35
Zuo M (2024)	RCS	HAIC+Apa+Cam	207	≤65/>65:190/17	185/22	B/C:15/192	187/20	121/86
		Apa+Cam	209	≤65/>65:181/28	188/21	B/C:34/175	209/0	47/162
Wang X (2024)	PCS	HAIC+Len+PD-1	33	NA	NA	C:33	NA	NA
		Len+PD-1	33	NA	NA	C:33	NA	NA
Wang D (2024)	RCS	HAIC+TKI+PD-1	99	56.40 ± 8.70	81/18	NA	70/29	34/65
		TKI+PD-1	95	57.11 ± 8.60	78/17	NA	72/23	24/71
Li R (2024)	RCS	HAIC+Len+PD-1	118	≤60/>60:102/16	103/15	B/C:9/109	NA	65/53
		Len+PD-1	114	≤60/>60:97/17	93/21	13/101	NA	59/55
Guan R (2024)	RCS	HAIC+Len+PD-1	127	51.90 ± 10.88	107/20	C:127	104/23	0/127
		Len+PD-1	103	54.01 ± 11.54	94/9	C:103	84/19	0/103
Diao L (2024)	RCS	HAIC+Len+PD-1	58	≤50/>50:16/52	49/9	24/34	49/9	39/19
		Len+PD-1	63	≤50/>50:5/27	50/13	25/38	55/8	42/21
Chang X (2024)	RCS	HAIC+Len+PD-1	103	52.0 ± 8.82	91/12	4/99	84/19	71/32
		Len+PD-1	61	56.0 ± 7.88	57/4	2/59	50/11	41/20
Fu Y (2023)	RCS	HAIC+Len+PD-1	89	51.9 ± 10.5	83/6	C:89	88/1	68/21
		Len+PD-1	53	53.5 ± 10.5	50/3	C:53	47/6	27/26
Mei J (2021)	RCS	HAIC+Len+PD-1	45	49.1 ± 10.6	38/7	5/40	44/1	30/15
		Len+PD-1	25	50.1 ± 12.3	18/7	3/22	22/3	12/13
Chen S (2021)	RCS	HAIC+Len+Pem	84	52(42-67)	72/12	22/62	71/13	64/20
		Len+Pem	86	53(43-69)	71/15	21/65	75/11	62/24

Apa, apatinib; BCLC, Barcelona Clinic Liver Cancer; Cam, camrelizumab; HAIC, hepatic arterial infusion chemotherapy; Len, lenvatinib; NA, not available; PCS, prospective cohort study; PD-1, programmed cell death-1; Pem, pembrolizumab; RCS, retrospective cohort study; SD, standard deviation; TKI, tyrosine kinase inhibitor.

### Overall survival

All 12 included studies provided data on OS. The aggregated survival data indicated a robust mortality risk reduction for patients receiving the triplet combination. Specifically, the pooled HR for OS in the H-T-P cohort was 0.51 (95% CI, 0.47-0.56; *P* < 0.001) relative to the T-P cohort (**Figure 2A**). No significant heterogeneity was observed among the studies (*I*^2^ = 0.0%; *P* = 0.936).

**Figure 2:**
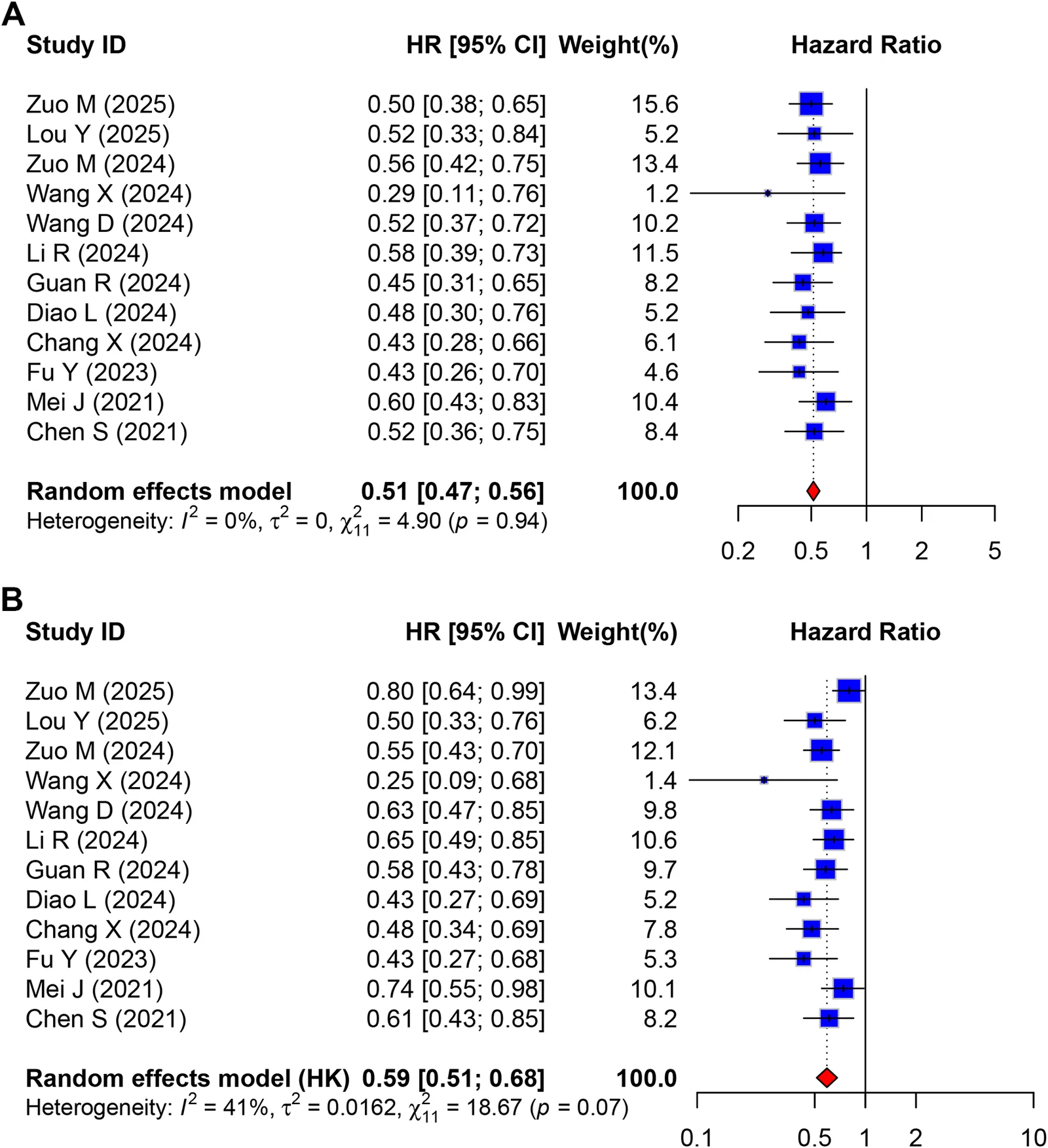
Forest plots comparing survival outcomes for triple versus dual therapy: **(A)** overall survival (OS) and **(B)** progression-free survival (PFS). CI, confidence interval; HR, hazard ratio.

### Progression-free survival

Data on PFS were reported in all included studies. Our meta-analysis confirmed that integrating HAIC into the TKI and PD-1 inhibitor backbone substantially delayed disease progression. The pooled HR was 0.59 (95% CI, 0.51-0.68; *P* < 0.001) in favor of the H-T-P regimen (**Figure 2B**). The analysis for heterogeneity indicated a moderate level of variation among studies (*I*^2^ = 41.1%), although Cochran’s Q test did not reach statistical significance (*P* = 0.067).

### Objective response rate and disease control rate

ORR data were available from all included studies. Patients treated with the H-T-P regimen achieved a nearly twofold increase in the likelihood of objective tumor response compared to those receiving dual therapy (RR, 1.97; 95% CI, 1.73-2.24; *P* < 0.001) (**Figure 3A**). A low degree of heterogeneity was present (*I*^2^ = 18.6%; *P* = 0.261). Similarly, with data from 11 studies, the pooled DCR was statistically superior in the triplet arm (RR, 1.19; 95% CI, 1.11-1.28; *P* < 0.001), with moderate heterogeneity observed (*I*^2^ = 42.2%; *P* = 0.068) (**Figure 3B**).

**Figure 3:**
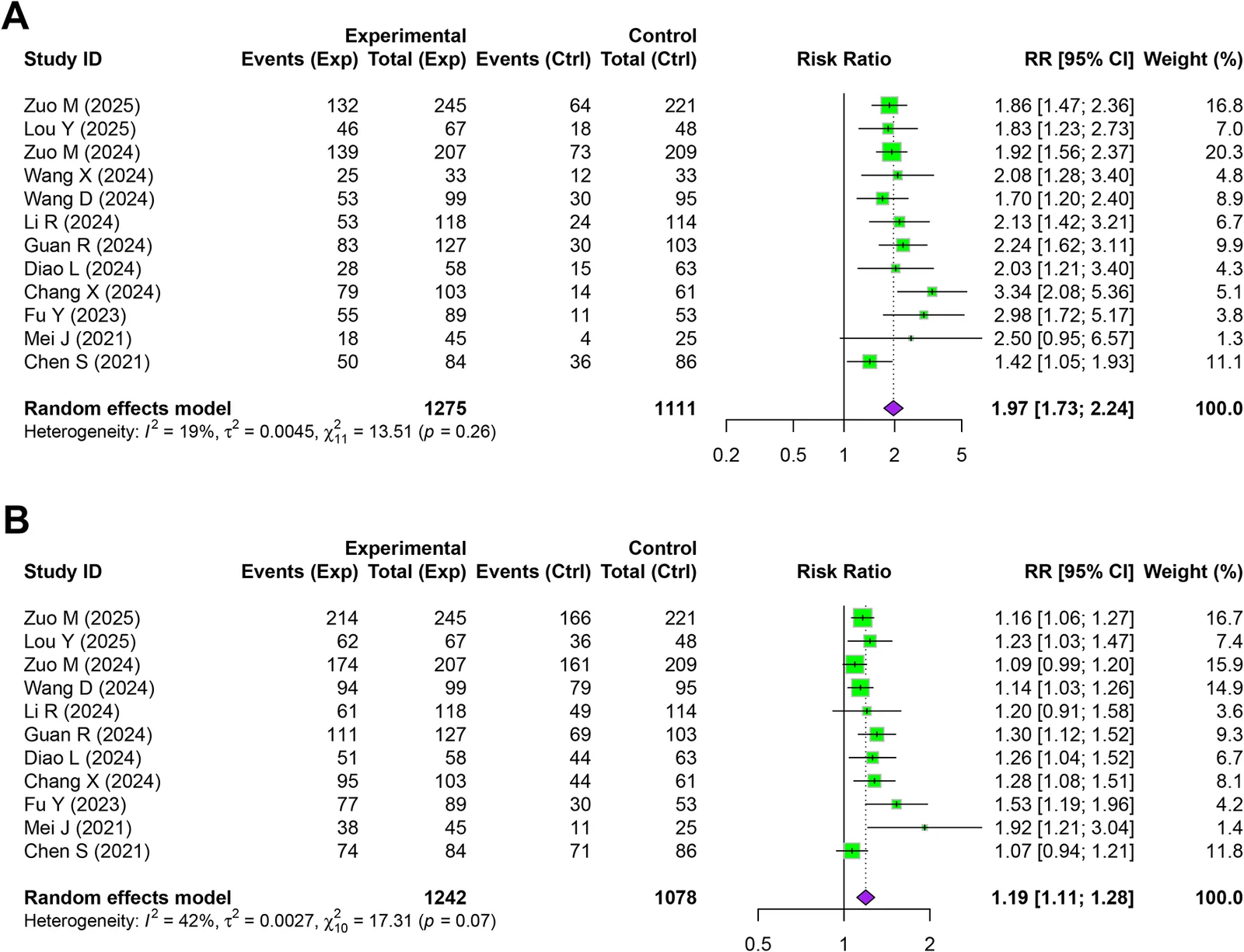
Forest plots comparing tumor response rates for triple versus dual therapy: **(A)** objective response rate (ORR) and **(B)** disease control rate (DCR). CI, confidence interval; RR, risk ratio.

### Prognostic factor analysis

A pooled analysis of prognostic factors was conducted for OS and PFS (**Table 2**). On multivariable analysis, the treatment regimen (triplet vs. doublet), Child-Pugh class (B vs. A), tumor number (>3 vs. ≤3), and the presence of liver cirrhosis were identified as independent predictors of OS. Furthermore, the presence of distant metastasis, liver cirrhosis, and the treatment regimen (triplet vs. doublet) were found to be independent factors significantly influencing PFS.

**Table 2:** Pooled multivariate analysis of prognostic factors for survival outcomes.

Factor	OS		PFS	
	HR [95% CI]	*P*	HR [95% CI]	*P*
Child-Pugh class (B/A)	2.57 [1.76, 3.73]	**<0.001**	1.67 [0.01, 304.24]	0.430
Distant Metastasis (present/absent)	1.59 [0.96, 2.64]	0.055	1.40 [1.03, 1.92]	**0.039**
BCLC stage (C/B)	1.48 [0.35, 6.24]	0.358	1.42 [0.56, 3.62]	0.466
Tumor number (>3/≤3)	1.69 [1.35, 2.11]	**0.022**	1.49 [0.90, 2.48]	0.063
Tumor number (multiple/single)	2.01 [0.06, 72.96]	0.245	1.53 [0.97, 2.52]	0.081
Tumor size, cm (≥10/<10)	1.00 [0.98, 1.02]	0.946	1.26 [0.92, 1.73]	0.152
Liver cirrhosis (yes/no)	1.60 [1.53, 1.66]	**0.004**	1.72 [1.05, 2.86]	**0.034**
PVTT (yes/no)	2.29 [0.83, 6.31]	0.072	1.81 [0.97, 3.38]	0.055
ALBI grade (≥II/I)	1.88 [0.25, 13.92]	0.155	1.26 [0.87, 1.84]	0.225
Treatment options (triple/dual)	0.44 [0.39, 0.50]	**<0.001**	0.51 [0.41, 0.63]	**<0.001**

ALBI, albumin-bilirubin; BCLC, Barcelona Clinic Liver Cancer; CI, confidence interval; OS, overall survival; PFS, progression-free survival; PVTT, portal vein tumor thrombus; HR, hazard ratio. Bold values indicate statistically significant results.

### Adverse events

Data on AEs were extracted from 11 studies. Analysis of the overall safety profile revealed no statistically significant difference in the overall incidence of all-grade AEs between the H-T-P and T-P arms (RR, 1.07; 95% CI, 0.92-1.25; *P* = 0.238), although heterogeneity was high (*I*^2^ = 71.5%; *P* = 0.015). In contrast, the incidence of grade ≥3 AEs was significantly higher in the H-T-P group (RR, 1.29; 95% CI, 1.13-1.48; *P* = 0.004), with no significant heterogeneity (*I*^2^ = 0.0%; *P* = 0.519).

A summary of specific AEs is provided in **Table 3**, categorized by affected organ systems. The most common AEs included elevated alanine aminotransferase (ALT) and aspartate aminotransferase (AST), hyperbilirubinemia, neutropenia, thrombocytopenia, abdominal pain, vomiting, diarrhea, hypertension, rash, and hand-foot syndrome. Elevated ALT and AST, neutropenia, thrombocytopenia, abdominal pain, and vomiting were more frequently observed in patients receiving H-T-P therapy, whereas the incidences of hyperbilirubinemia, diarrhea, hypertension, rash, and hand-foot syndrome did not differ significantly between the two groups.

**Table 3:** Comparison of treatment-related adverse events between the triple-therapy and dual-therapy arms.

Adverse events	All grade		Grade ≥3	
	RR [95% CI]	*P*	RR [95% CI]	*P*
Elevated ALT	1.77 [1.29; 2.42]	**0.003**	1.97 [1.42, 2.74]	**0.001**
Elevated AST	1.70 [1.22, 2.38]	**0.006**	2.01 [1.31, 3.08]	**0.006**
Hyperbilirubinemia	1.41 [0.95, 2.10]	0.078	1.61 [0.75, 3.43]	0.183
Hypoalbuminemia	1.05 [0.88, 1.26]	0.490	1.42 [1.03, 1.95]	0.041
Anemia	2.13 [1.55, 2.91]	**0.001**	1.88 [1.17, 3.01]	**0.019**
Neutropenia	2.56 [1.44, 4.55]	**0.005**	3.86 [3.02, 4.94]	**<0.001**
Thrombocytopenia	1.90 [1.12, 3.22]	**0.023**	2.08 [1.60, 2.71]	**<0.001**
Hypothyroidism	0.98 [0.70, 1.38]	0.897	0.97 [0.16, 5.96]	0.965
Elevated creatinine	1.71 [1.26, 2.32]	**0.007**	2.61 [0.78, 8.74]	0.063
Proteinuria	1.56 [1.13, 2.17]	**0.014**	0.83 [0.48, 1.42]	0.424
Fever	2.36 [1.17, 4.76]	**0.024**	1.25 [0.19, 8.05]	0.657
Fatigue	1.30 [1.07, 1.58]	**0.012**	1.23 [0.58, 2.61]	0.522
Abdominal pain	2.57 [1.23, 5.38]	**0.018**	4.26 [1.33, 13.62]	**0.023**
Nausea	1.92 [1.13, 3.25]	**0.023**	2.27 [0.38, 13.50]	0.238
Vomiting	3.42 [1.80, 6.48]	**0.003**	5.71 [3.16, 10.31]	**<0.001**
Diarrhea	1.12 [0.83, 1.51]	0.423	1.59 [0.72, 3.51]	0.208
Decreased appetite	1.30 [0.96, 1.77]	0.081	1.92 [0.81, 4.55]	0.094
Hoarseness	0.79 [0.45, 1.36]	0.202	0.17 [0.06, 0.44]	**0.027**
Rash	1.47 [0.95, 2.25]	0.075	2.01 [0.74, 5.43]	0.139
Pruritus	1.28 [0.69, 2.37]	0.301	4.80 [0.23, 98.67]	0.309
Allergy	0.73 [0.12, 4.65]	0.278	1.79 [0.07, 43.24]	0.719
HFS	1.05 [0.39, 2.86]	0.876	1.08 [0.36, 3.25]	0.839
Oral ulcer	0.97 [0.09, 10.83]	0.915	1.06 [0.57, 1.97]	0.439
Weight loss	1.26 [0.76, 2.10]	0.188	1.01 [0.05, 19.14]	0.992
Hypertension	1.01 [0.83, 1.23]	0.926	0.90 [0.45, 1.79]	0.722
Hepatitis	0.96 [0.45, 2.04]	0.889	0.47 [0.13, 1.71]	0.162
Pneumonia	0.75 [0.27, 2.05]	0.468	0.81 [0.12, 5.59]	0.749

ALT, alanine aminotransferase; AST, aspartate aminotransferase; CI, confidence interval; HFS, hand-foot syndrome; RR, risk ratio. Bold values indicate statistically significant results.

### Publication bias and sensitivity analysis

For OS, the funnel plot exhibited some asymmetry (**Supplementary Figure 1A**). Egger’s test confirmed the presence of this asymmetry (*P* = 0.011), suggesting potential publication bias. To assess the impact of this bias on the robustness of the pooled result, a trim-and-fill analysis was performed. This method imputed three potentially missing studies into the model (**Supplementary Figure 2A**), yielding an adjusted HR of 0.53 (95% CI, 0.49-0.58; *P* < 0.001). This estimate was only slightly altered from the original effect size (HR = 0.51) and remained highly statistically significant, indicating that the finding for OS is robust. Similarly, the assessment for PFS revealed significant funnel plot asymmetry (**Supplementary Figure 1B**), which was supported by Egger’s test (*P* = 0.002). The trim-and-fill analysis, after imputing four hypothetical studies (**Supplementary Figure 2B**), produced an adjusted HR of 0.65 (95% CI, 0.56-0.76; *P* < 0.001). As this did not reverse the original conclusion, the robustness of the PFS result was confirmed.

For ORR, the funnel plot appeared largely symmetrical (**Supplementary Figure 1C**), and Harbord’s test detected no significant publication bias (*P* = 0.748). In contrast, the assessment for DCR suggested significant publication bias (Harbord’s test, *P* = 0.014) (**Supplementary Figure 1D**). After adjusting for this bias using the trim-and-fill method (**Supplementary Figure 2C**), the advantage of triple therapy in improving DCR persisted, with a pooled RR of 1.14 (95% CI, 1.04-1.24; *P* = 0.007), affirming the robustness of this conclusion.

Finally, a leave-one-out sensitivity analysis was conducted for all primary outcomes. The results showed that the sequential removal of any single study did not substantially alter the pooled effect estimates (**Supplementary Figure 3**), further demonstrating the stability and reliability of the meta-analytic conclusions.

## Discussion

The present study represents the most comprehensive meta-analysis to date comparing the triple-therapy regimen of HAIC, a TKI, and a PD-1 inhibitor against the dual-therapy combination of a TKI and a PD-1 inhibitor for the treatment of uHCC. While previous meta-analyses have explored the combination of systemic therapy with locoregional treatments, they often grouped HAIC with transarterial chemoembolization (TACE) (41). Our study uniquely focuses exclusively on the HAIC-based triplet regimen, thereby eliminating the confounding effects of different local therapies and providing a more precise, homogeneous evaluation of its distinct clinical value. Our findings provide compelling evidence that, in a large cohort of over 2,300 patients, the H-T-P triple-therapy is markedly superior to T-P dual-therapy in prolonging both OS and PFS. Furthermore, the triple therapy yielded a nearly twofold higher ORR and an improved DCR.

A central finding of our analysis is the substantial survival advantage conferred by the triple therapy. The pooled HR for OS was 0.51, corresponding to a nearly 50% reduction in the risk of death—an improvement of profound clinical significance. The significant extension of PFS (HR, 0.59) further corroborates the robust efficacy of the triplet regimen in maintaining durable disease control. These results are broadly consistent with those of another meta-analysis that included both TACE and HAIC, which similarly affirmed the superiority of combining locoregional and systemic therapies, reporting an OS HR of 0.47 and a PFS HR of 0.52 (41). This survival benefit is likely attributable to the synergistic interplay of the different treatment modalities (15). As a highly effective local debulking strategy, HAIC can rapidly reduce tumor burden and induce immunogenic cell death. This is complemented by the TKI, which inhibits angiogenesis and modulates the tumor microenvironment to enhance the immune response, while the PD-1 inhibitor serves to unleash a systemic anti-tumor T-cell response by overcoming immune suppression. The combination thereby achieves an effective integration of locoregional control and systemic therapy (13, 15).

Another key finding of this study is the exceptional performance of the triple therapy in terms of tumor response. The pooled RR for ORR reached 1.97, indicating that the triplet regimen’s capacity to induce tumor shrinkage far exceeds that of the dual-therapy regimen. Such a high response rate not only alleviates tumor-related symptoms but, more importantly, creates an opportunity for conversion to curative-intent surgery in patients who were initially unresectable—a strategy known as conversion therapy (15, 39, 42). The clinical significance of this conversion strategy is profoundly underscored by the evolving landscape of perioperative care. Very recent updated data from the phase 3 IMbrave050 trial demonstrated that adjuvant atezolizumab plus bevacizumab failed to sustain an early recurrence-free survival benefit after curative resection or ablation (43). Given the current lack of proven, highly effective systemic adjuvant therapies to prevent postoperative relapse, achieving a deep and robust initial tumor response—and potentially complete pathological downstaging—through a potent H-T-P conversion regimen prior to surgery becomes absolutely critical for maximizing long-term survival.

Regarding safety, our analysis found that while the rate of grade ≥3 adverse events was higher in the triple-therapy group, the overall incidence of all-grade AEs did not differ significantly from the dual-therapy arm. It is critical to distinguish the sources of these toxicities to guide clinical management. The increased incidence of severe AEs in the triplet arm was primarily driven by HAIC-specific toxicities, such as hepatic dysfunction (elevated ALT/AST), myelosuppression (neutropenia, thrombocytopenia), and procedure-related abdominal pain and vomiting. In contrast, systemic toxicities typical of TKIs and PD-1 inhibitors—such as hypertension, hand-foot syndrome, and immune-related adverse events (e.g., rash, diarrhea)—remained comparable between the two groups. In clinical practice, these HAIC-induced severe toxicities are highly predictable and manageable. Hepatotoxicity can be effectively addressed with transient dose adjustments, delaying the next HAIC cycle, and administering hepatoprotective agents. Similarly, myelosuppression can be safely managed using granulocyte colony-stimulating factor (G-CSF), thrombopoietin receptor agonists (TPO-RAs), or timely blood transfusions (14). Therefore, with oversight from an experienced multidisciplinary team, the addition of HAIC does not unacceptably exacerbate the toxicity profile.

The first-line standard of care for uHCC is currently transitioning from TKI monotherapy toward immunotherapy-based combination regimens (5, 44). Both the “ICI + anti-angiogenic” combination (e.g., atezolizumab plus bevacizumab) and dual immunotherapy (e.g., durvalumab plus tremelimumab) have received top-level recommendations in international guidelines (44). However, these systemic doublet regimens often yield limited objective response rates (typically around 20-30%). The H-T-P regimen, by integrating potent locoregional tumor debulking, achieves significantly higher response rates. This makes the triplet combination uniquely advantageous and complementary to current treatment algorithms, particularly as a conversion therapy for patients with a massive tumor burden or portal vein tumor thrombosis (PVTT) who might not achieve sufficient cytoreduction with systemic doublets alone.

Furthermore, the role of this triplet regimen in the context of prior ICI exposure remains an open question. Due to data limitations in the original studies, we could not perform a subgroup analysis stratified by previous ICI treatment. However, as ICI rechallenge is increasingly explored in HCC, evaluating whether the immunomodulatory effects of HAIC can resensitize tumors to PD-1 inhibitors in ICI-refractory patients represents a crucial area for future research (45).

The strengths of this study include its rigorous adherence to the PRISMA guidelines and its prospective PROSPERO registration. By including a large number of patients, our analysis has high statistical power. Moreover, we employed robust statistical methods, including a random-effects model and comprehensive sensitivity analyses (e.g., the trim-and-fill method and leave-one-out analysis), which confirmed that the conclusions of our meta-analysis are both reliable and stable.

Nevertheless, this study has several limitations. First, the predominance of retrospective cohort studies introduces inherent risks of selection bias, unmeasured confounding, and missing data, which may skew the pooled estimates. Patients receiving triple therapy in retrospective settings might possess better baseline performance status or liver function, thus influencing the results. Second, there are inherent efficacy and safety differences among the various TKIs (e.g., lenvatinib vs. sorafenib) and PD-1 inhibitors (e.g., camrelizumab vs. sintilimab) utilized across the included studies. Grouping these distinct pharmacological agents together inevitably introduces clinical confounding. Third, our analysis detected publication bias for OS, PFS, and DCR. Although trim-and-fill analyses confirmed the stability of our findings, the presence of these biases and moderate heterogeneity implies that the overall effect sizes might be slightly overestimated. Clinicians should interpret these pooled benefits with caution, acknowledging that real-world outcomes may vary based on institutional experience and patient selection. Finally, the lack of individual patient data precluded our ability to conduct more granular analyses of specific subgroups, such as by BCLC stage or the presence of vascular invasion.

## Conclusion

In conclusion, this meta-analysis furnishes the most comprehensive evidence to date comparing H-T-P triple therapy with T-P dual therapy. Our findings demonstrate that for patients with uHCC, the incorporation of HAIC into a regimen with a TKI and a PD-1 inhibitor leads to significant improvements in survival outcomes and tumor response rates. This enhanced efficacy is achieved with a predictable and clinically manageable safety profile. The H-T-P regimen, therefore, represents a compelling therapeutic option for uHCC, particularly for patients with a high tumor burden who are candidates for conversion to curative treatment. Looking forward, large-scale, multicenter RCTs are imperative to definitively confirm the superiority of this triple-therapy regimen. Future investigations should also focus on identifying biomarkers predictive of treatment efficacy and toxicity to enable patient stratification for personalized therapy. Furthermore, research is warranted to refine the optimal HAIC protocols, treatment timing, and the strategic integration of this locoregional therapy with systemic agents to maximize clinical benefit.

## Data Availability

The original contributions presented in the study are included in the article/**Supplementary Material**, further inquiries can be directed to the corresponding author/s.
