# Supplementary material for: Efficacy and safety of hepatic arterial infusion chemotherapy combined with tyrosine kinase inhibitors and PD-1 inhibitors for unresectable hepatocellular carcinoma: a meta-analysis
Source: Front Oncol. 2026 Oct 8;16:1896692. doi: 10.3389/fonc.2026.1896692 (PMC13686884; doi:10.3389/fonc.2026.1896692)
Supplement: Supplementary file 1 [file DataSheet1.pdf]

Supplementary Figures

(A)

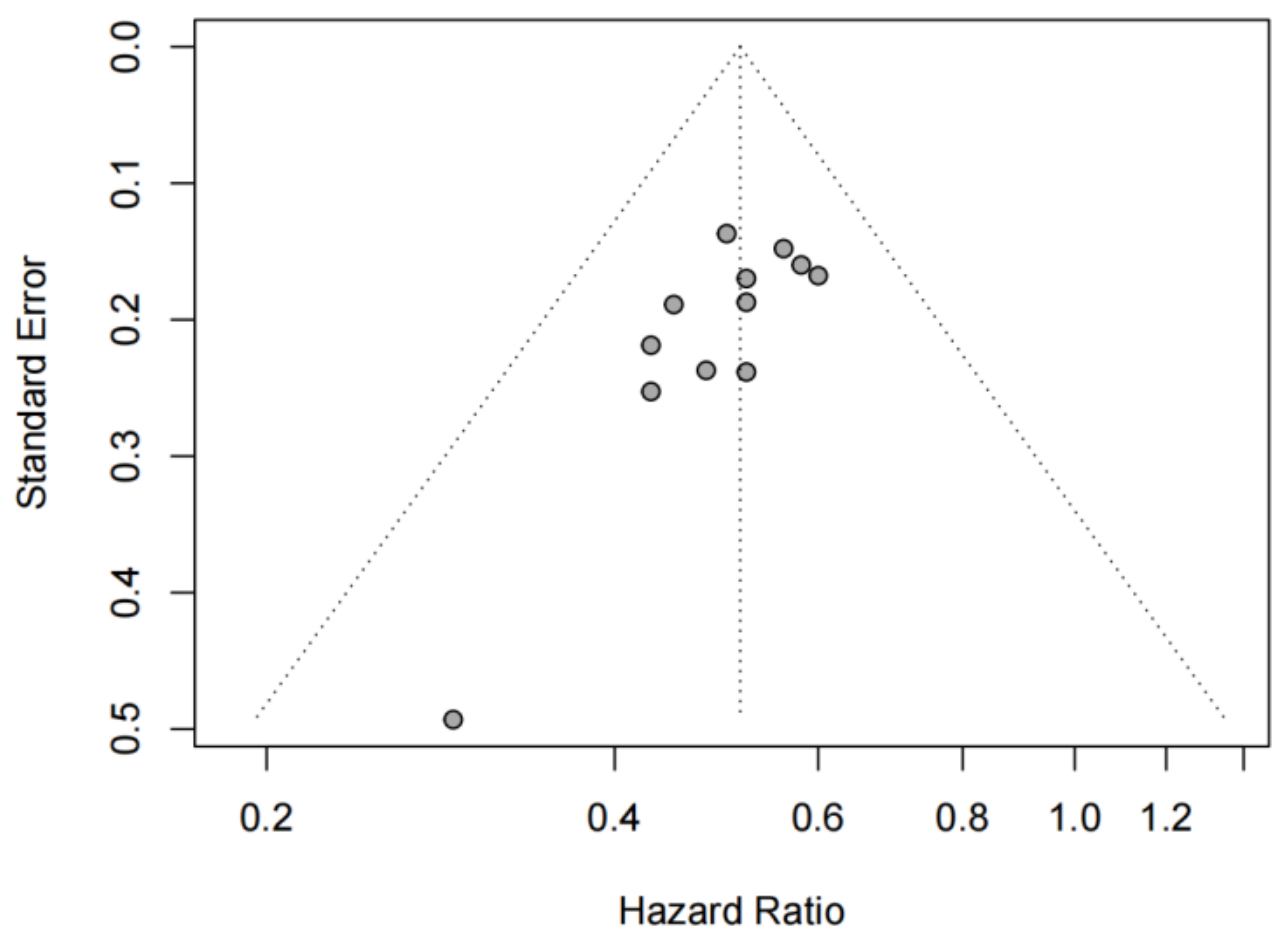

(B)

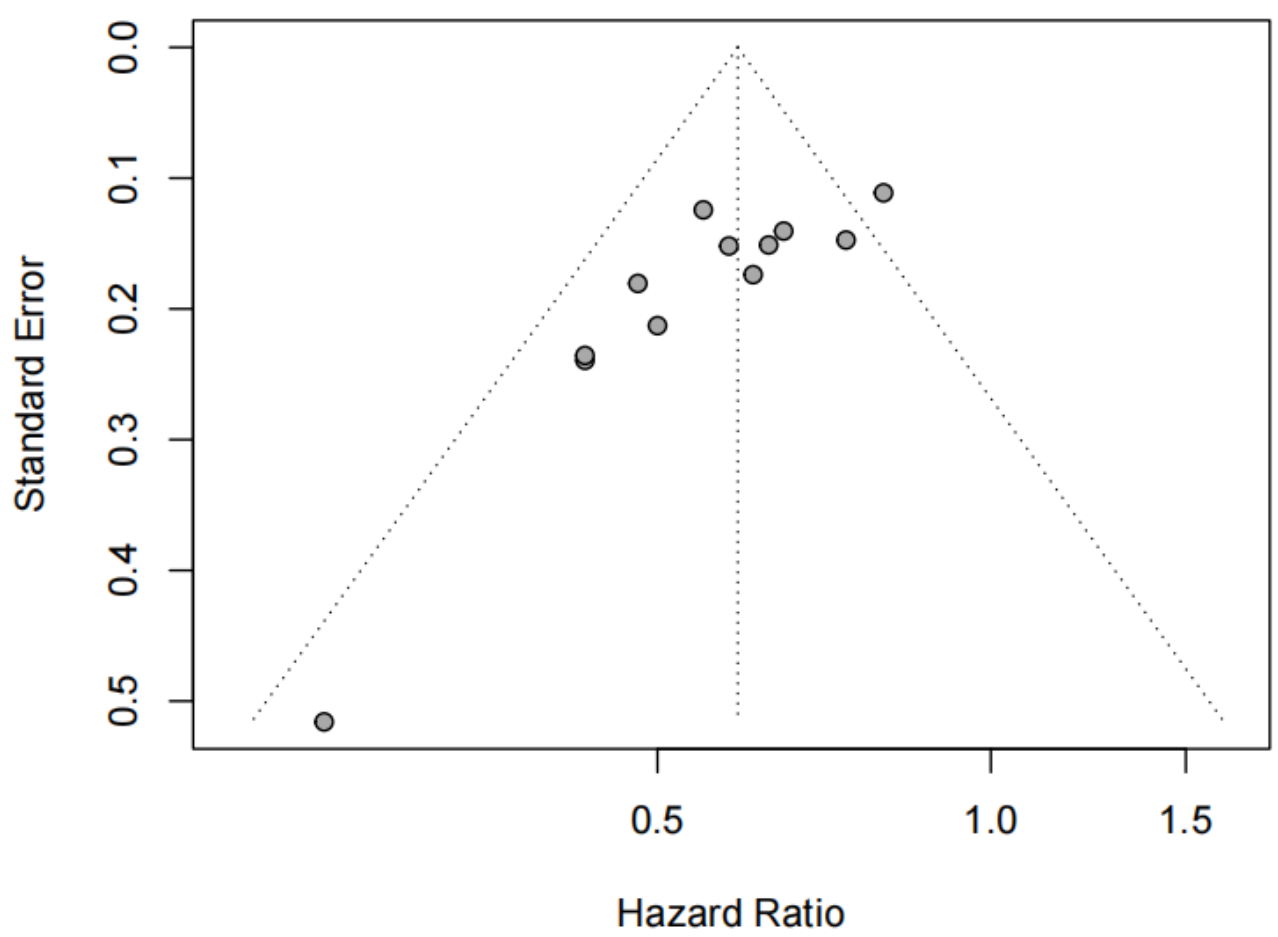

(C)

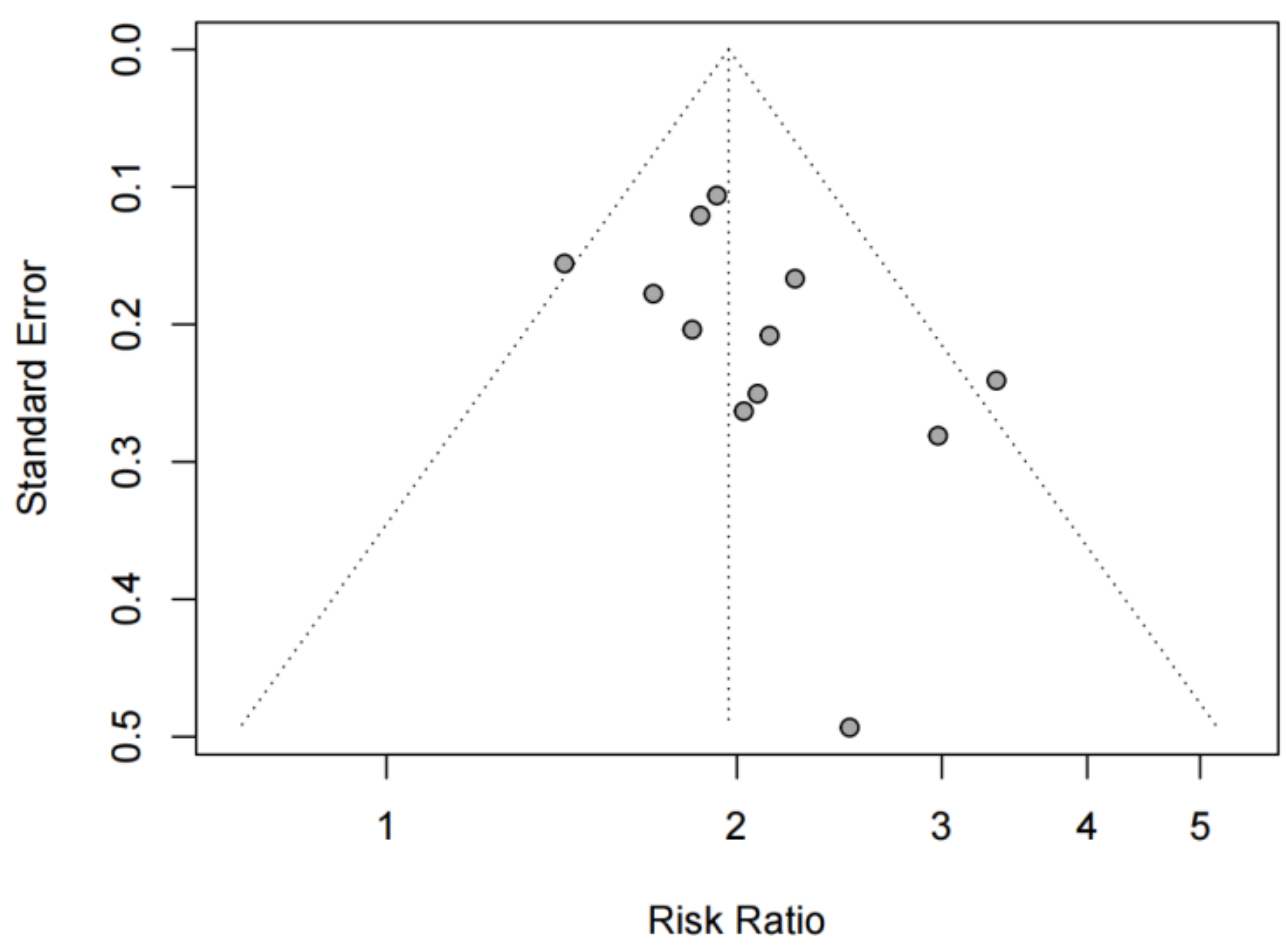

(D)

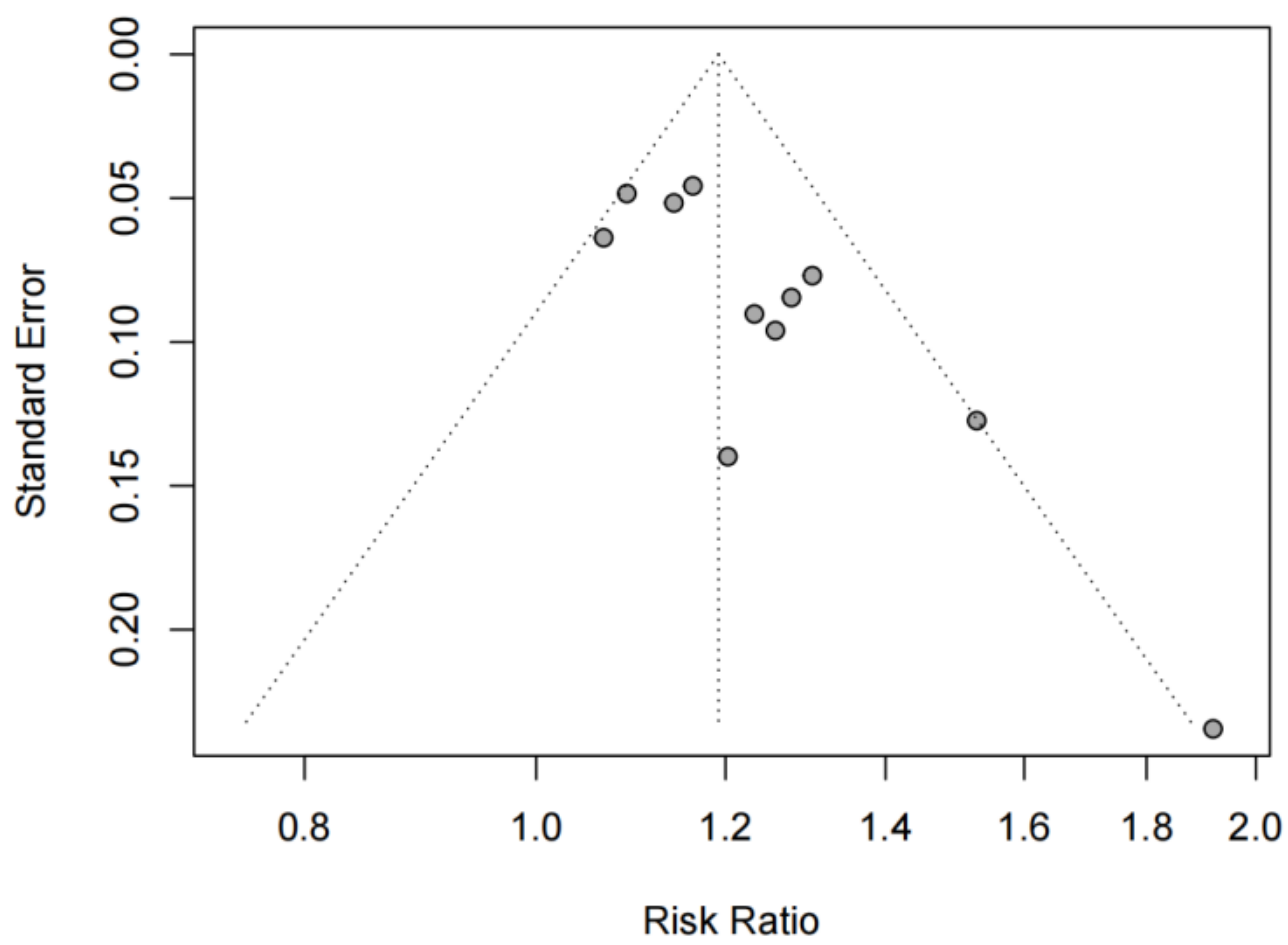

Supplementary Figure 1. Funnel plots of OS (A), PFS (B), ORR (C), and DCR(D) for checking potential publication bias.

(A)

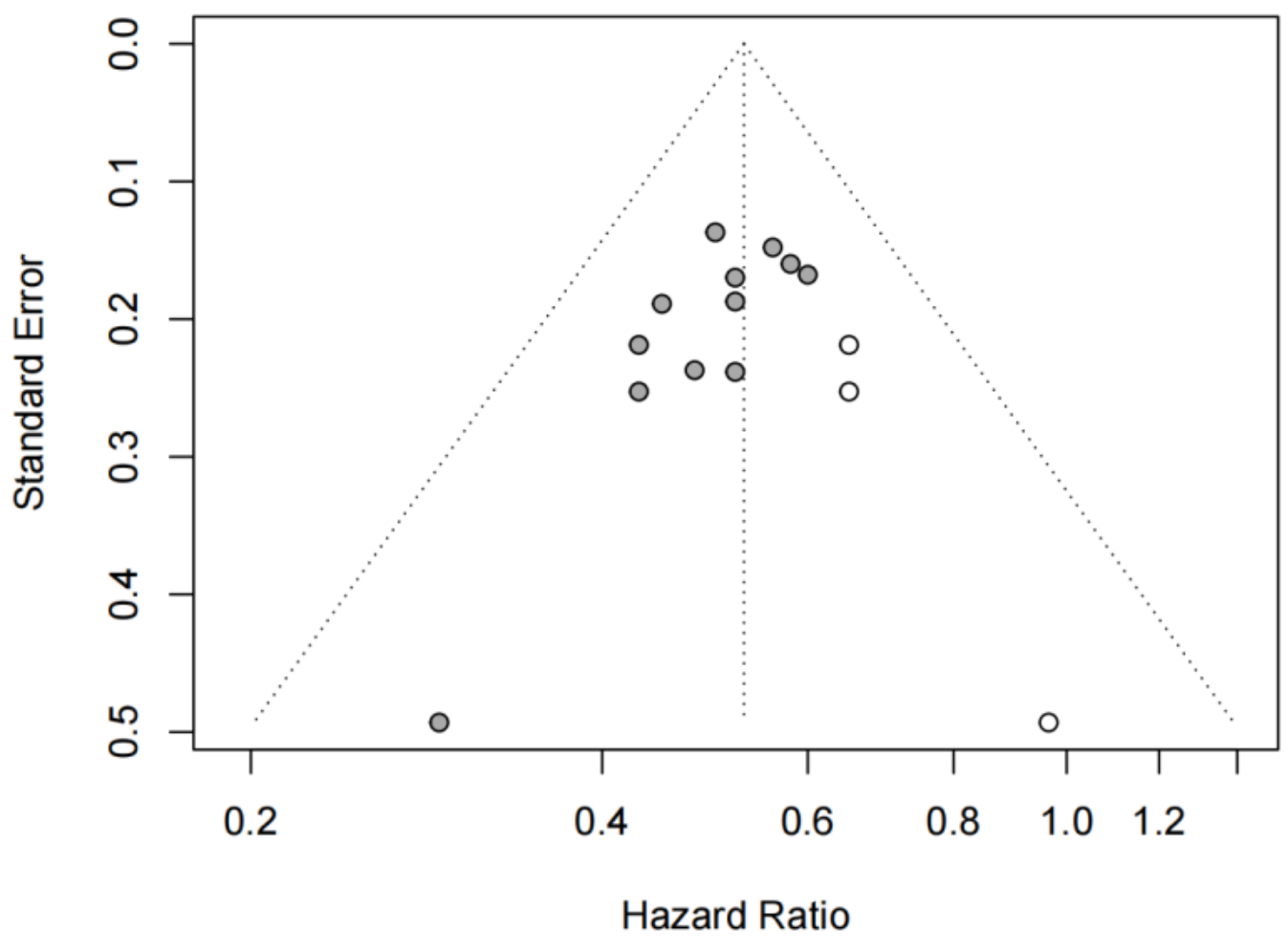

(B)

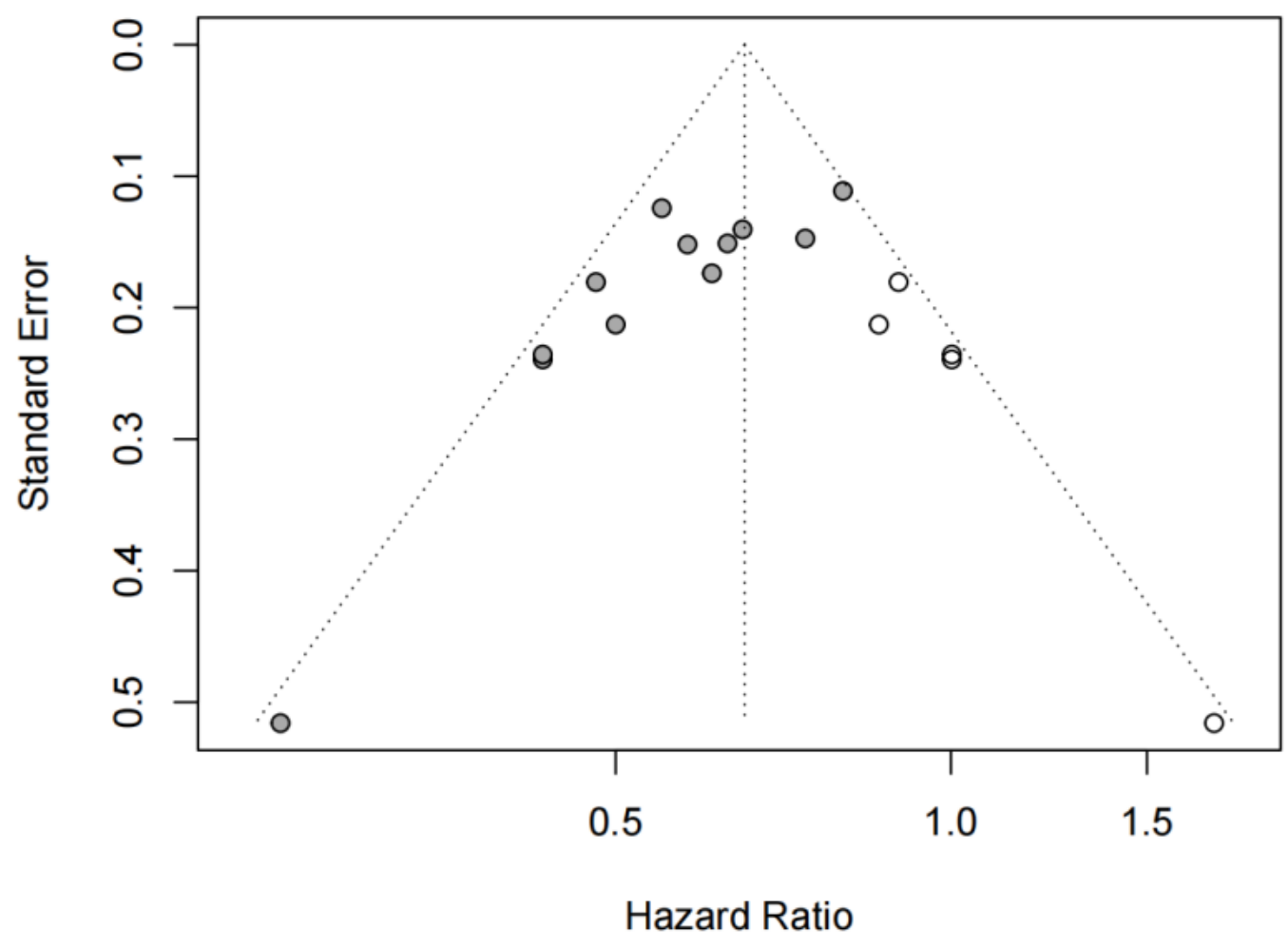

(C)

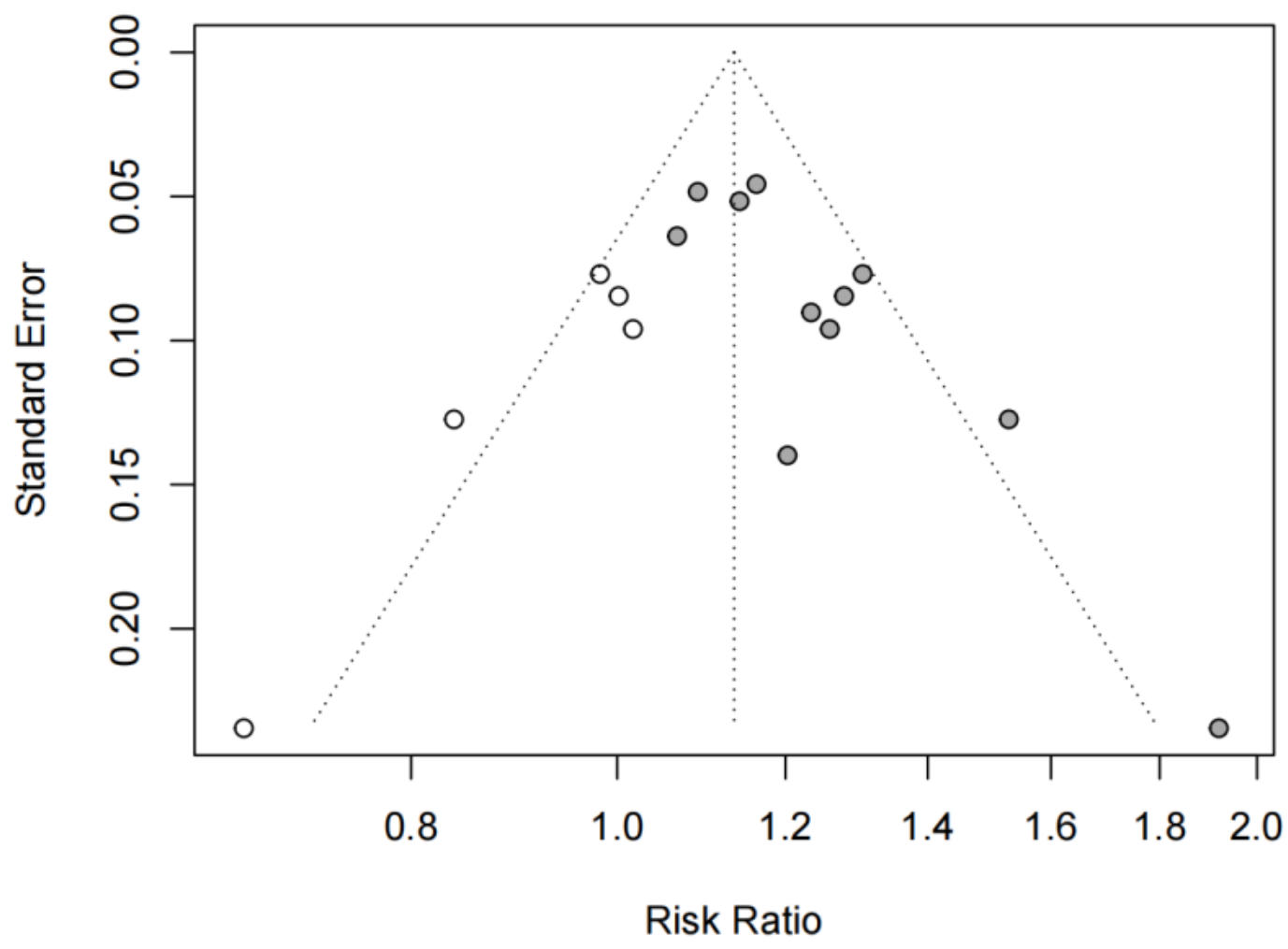

Supplementary Figure 2. Funnel plots of OS (A), PFS (B), and DCR (C) regenerated after filling the hypothetical study using the Trim and Fill method.

(A)

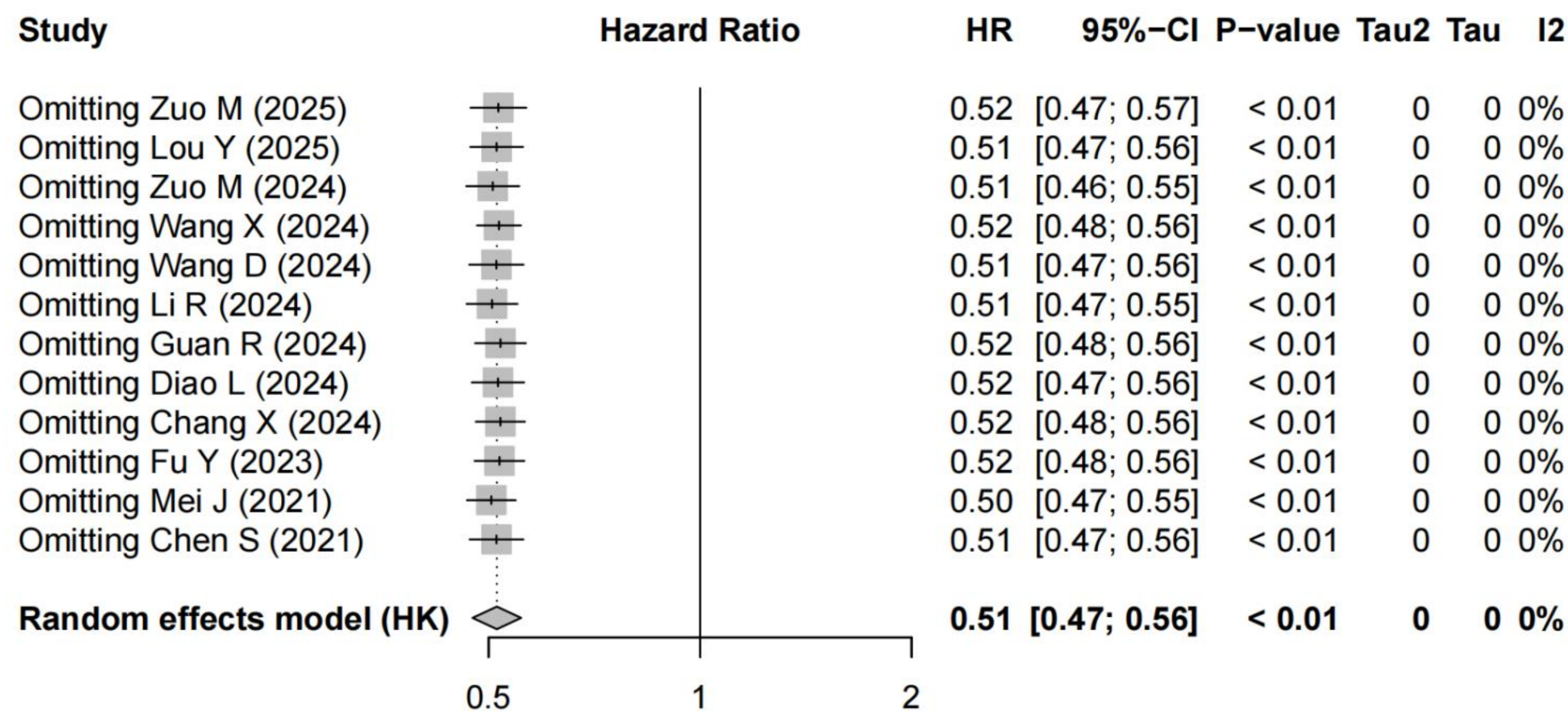
Study
Hazard Ratio
HR 95%−CI P−value Tau2 Tau I2
Omitting Zuo M (2025) 0.52 [0.47; 0.57] < 0.01 0 0 0%
Omitting Lou Y (2025) 0.51 [0.47; 0.56] < 0.01 0 0 0%
Omitting Zuo M (2024) 0.51 [0.46; 0.55] < 0.01 0 0 0%
Omitting Wang X (2024) 0.52 [0.48; 0.56] < 0.01 0 0 0%
Omitting Wang D (2024) 0.51 [0.47; 0.56] < 0.01 0 0 0%
Omitting Li R (2024) 0.51 [0.47; 0.55] < 0.01 0 0 0%
Omitting Guan R (2024) 0.52 [0.48; 0.56] < 0.01 0 0 0%
Omitting Diao L (2024) 0.52 [0.47; 0.56] < 0.01 0 0 0%
Omitting Chang X (2024) 0.52 [0.48; 0.56] < 0.01 0 0 0%
Omitting Fu Y (2023) 0.52 [0.48; 0.56] < 0.01 0 0 0%
Omitting Mei J (2021) 0.50 [0.47; 0.55] < 0.01 0 0 0%
Omitting Chen S (2021) 0.51 [0.47; 0.56] < 0.01 0 0 0%
Random effects model (HK) 0.51 [0.47; 0.56] < 0.01 0 0 0%
0.5
1
2

(B)

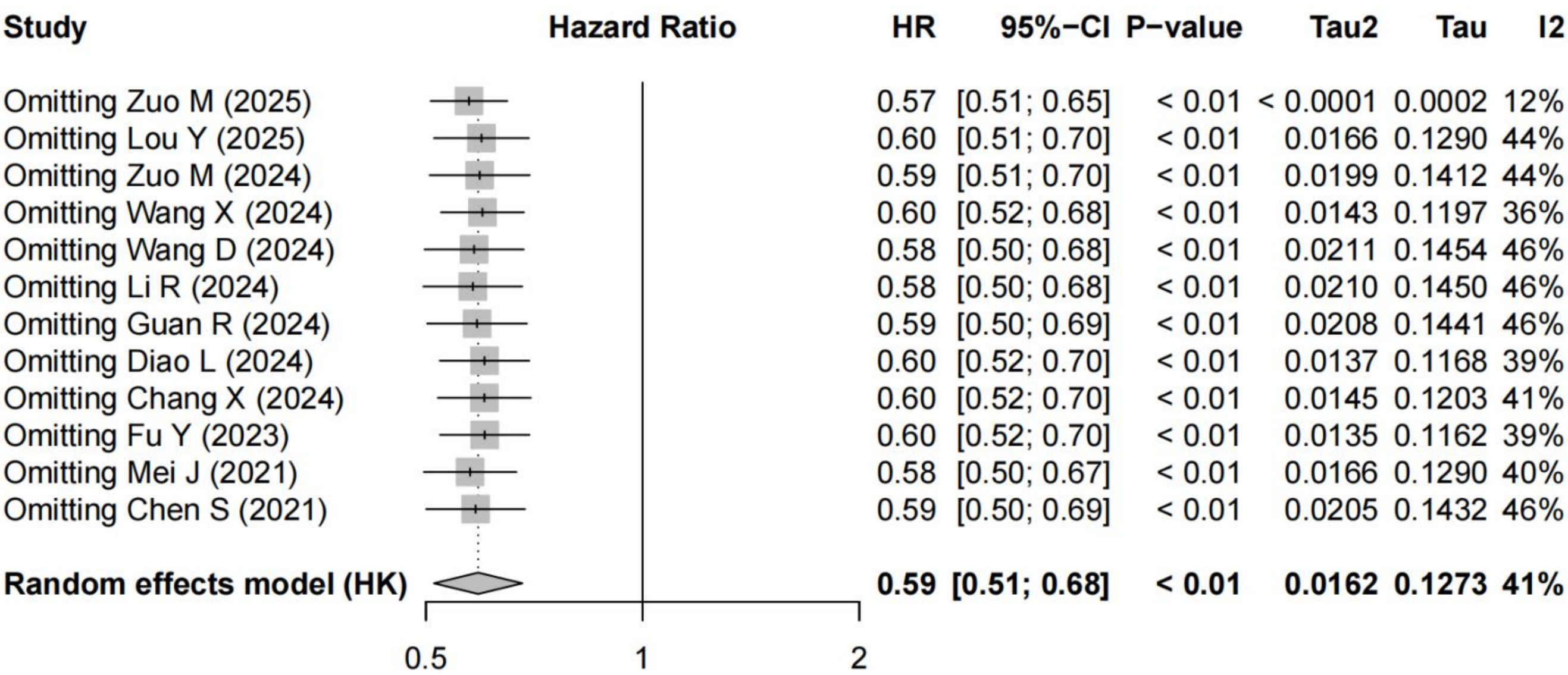
Study
Hazard Ratio
HR 95%−CI P−value Tau2 Tau I2
Omitting Zuo M (2025) 0.57 [0.51; 0.65] < 0.01 < 0.0001 0.0002 12%
Omitting Lou Y (2025) 0.60 [0.51; 0.70] < 0.01 0.0166 0.1290 44%
Omitting Zuo M (2024) 0.59 [0.51; 0.70] < 0.01 0.0199 0.1412 44%
Omitting Wang X (2024) 0.60 [0.52; 0.68] < 0.01 0.0143 0.1197 36%
Omitting Wang D (2024) 0.58 [0.50; 0.68] < 0.01 0.0211 0.1454 46%
Omitting Li R (2024) 0.58 [0.50; 0.68] < 0.01 0.0210 0.1450 46%
Omitting Guan R (2024) 0.59 [0.50; 0.69] < 0.01 0.0208 0.1441 46%
Omitting Diao L (2024) 0.60 [0.52; 0.70] < 0.01 0.0137 0.1168 39%
Omitting Chang X (2024) 0.60 [0.52; 0.70] < 0.01 0.0145 0.1203 41%
Omitting Fu Y (2023) 0.60 [0.52; 0.70] < 0.01 0.0135 0.1162 39%
Omitting Mei J (2021) 0.58 [0.50; 0.67] < 0.01 0.0166 0.1290 40%
Omitting Chen S (2021) 0.59 [0.50; 0.69] < 0.01 0.0205 0.1432 46%
Random effects model (HK) 0.59 [0.51; 0.68] < 0.01 0.0162 0.1273 41%
0.5
1
2

(C)

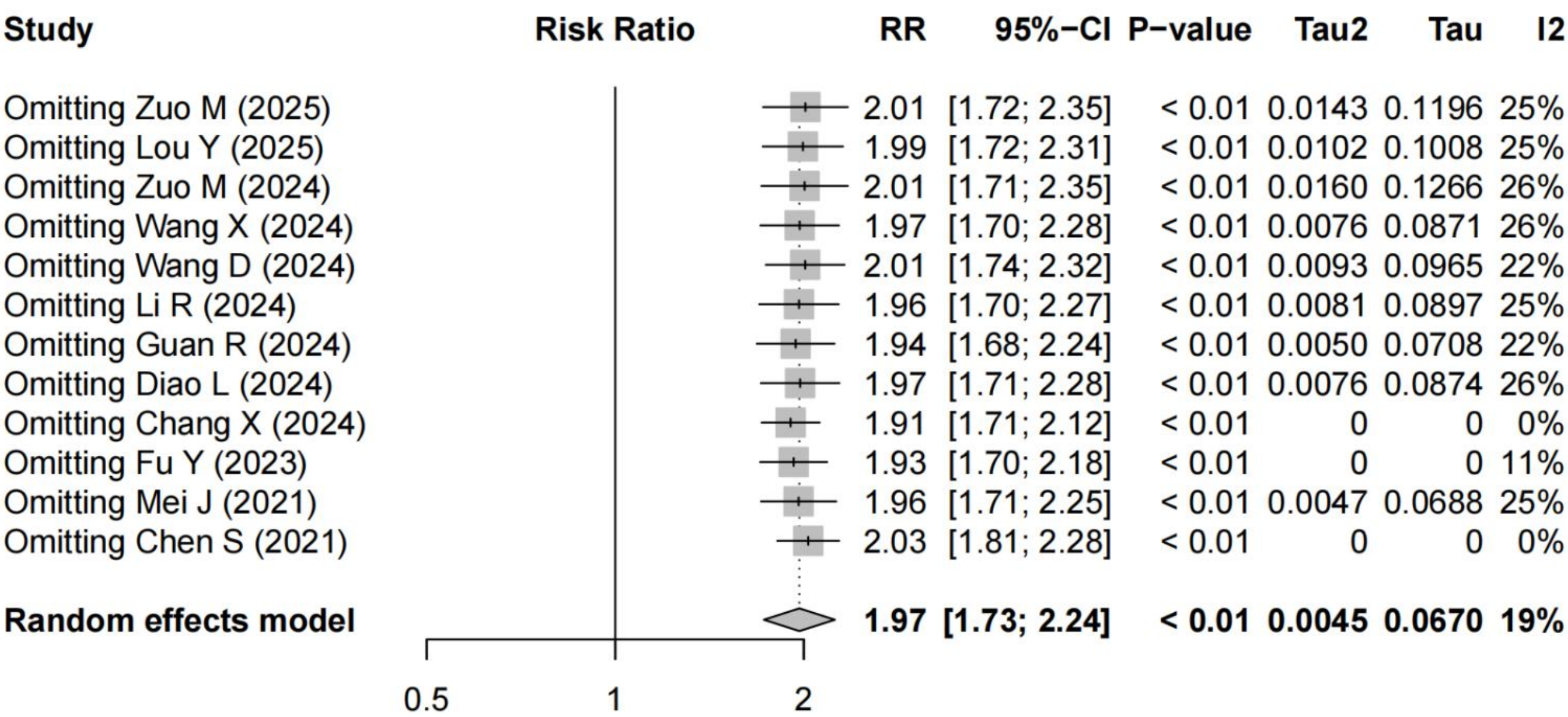
Study
Risk Ratio
RR 95%−CI P−value Tau2 Tau I2
Omitting Zuo M (2025) 2.01 [1.72; 2.35] < 0.01 0.0143 0.1196 25%
Omitting Lou Y (2025) 1.99 [1.72; 2.31] < 0.01 0.0102 0.1008 25%
Omitting Zuo M (2024) 2.01 [1.71; 2.35] < 0.01 0.0160 0.1266 26%
Omitting Wang X (2024) 1.97 [1.70; 2.28] < 0.01 0.0076 0.0871 26%
Omitting Wang D (2024) 2.01 [1.74; 2.32] < 0.01 0.0093 0.0965 22%
Omitting Li R (2024) 1.96 [1.70; 2.27] < 0.01 0.0081 0.0897 25%
Omitting Guan R (2024) 1.94 [1.68; 2.24] < 0.01 0.0050 0.0708 22%
Omitting Diao L (2024) 1.97 [1.71; 2.28] < 0.01 0.0076 0.0874 26%
Omitting Chang X (2024) 1.91 [1.71; 2.12] < 0.01 0 0 0%
Omitting Fu Y (2023) 1.93 [1.70; 2.18] < 0.01 0 0 11%
Omitting Mei J (2021) 1.96 [1.71; 2.25] < 0.01 0.0047 0.0688 25%
Omitting Chen S (2021) 2.03 [1.81; 2.28] < 0.01 0 0 0%
Random effects model 1.97 [1.73; 2.24] < 0.01 0.0045 0.0670 19%
0.5
1
2

(D)

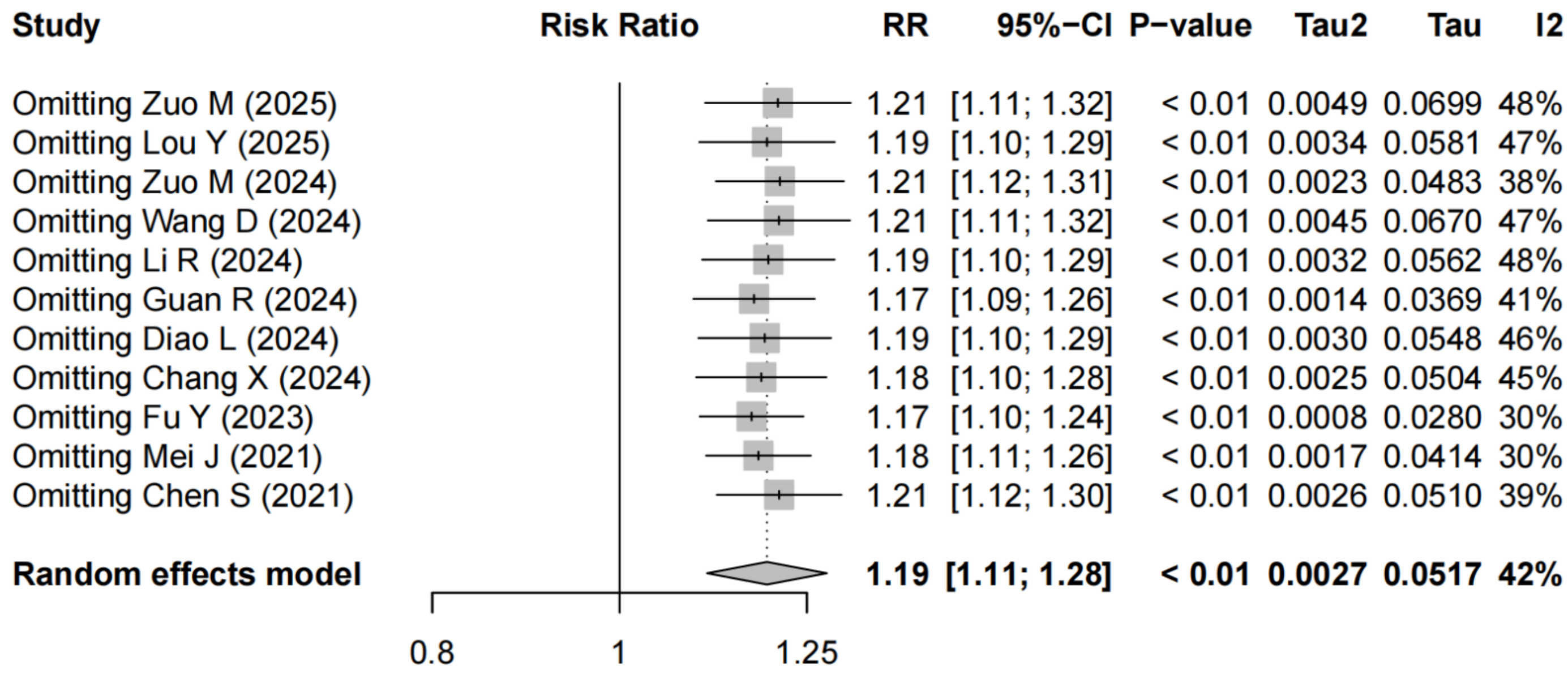

Supplementary Figure 3. Sensitivity analysis results of one-to-one elimination experiments. (A) OS; (B) PFS; (C) ORR; (D) DCR.
