# Supplementary material for: Efficacy and safety of hepatic arterial infusion chemotherapy combined with tyrosine kinase inhibitors and PD-1 inhibitors for unresectable hepatocellular carcinoma: a meta-analysis
Source: Front Oncol. 2026 Oct 8;16:1896692. doi: 10.3389/fonc.2026.1896692 (PMC13686884; doi:10.3389/fonc.2026.1896692)
Supplement: Supplementary file 2 [file DataSheet2.pdf]

Supplementary Table

Supplementary Table 1. Database search strategies

Search engines	EMBASE, PubMed, and the Cochrane Library
Search keywords	Hepatocellular carcinoma, liver cancer, HCC, liver cell carcinoma, programmed cell death-1, PD-1, programmed death-ligand 1, PD-L1, cytotoxic T-lymphocyte antigen-4, CTLA-4, immune checkpoint inhibitors, ICIs, tyrosine kinase inhibitor, TKI, hepatic arterial infusion chemotherapy, HAIC
Study population	Patients diagnosed with advanced or unresectable hepatocellular carcinoma
Inclusion criteria	(1) involved patients with advanced or unresectable hepatocellular carcinoma confirmed by pathological or radiological evidence<br>(2) the experimental group received triple therapy with HAIC, a TKI, and a PD-1 inhibitor<br>(3) the control group received dual therapy with a TKI plus a PD-1 inhibitor<br>(4) reported at least one of the following outcomes: overall survival, progression-free survival, objective response rate, disease control rate, or treatment-related adverse events<br>(5) were either randomized controlled trials (RCTs) or comparative cohort studies (prospective or retrospective)
Exclusion criteria	(1) reviews, case reports, letters, editorials, and commentaries<br>(2) studies with inappropriate interventions or comparators<br>(3) studies from which the necessary data could not be extracted<br>(4) duplicate publications, in which case the most comprehensive or recent report was retained.
Dates	From inception of the databases to July 2025
Geographical area	No geographic restrictions
Language	No restriction on language of publication
Search Strategy for PubMed	((((((Hepatocellular carcinoma[MeSH Terms]) OR (Hepatocellular carcinoma[Title/Abstract])) OR (HCC[Title/Abstract])) OR (liver

	cancer[Title/Abstract])) OR (Liver Cell Carcinoma[Title/Abstract])) AND ((((((((((((Immunotherapy[Title/Abstract]) OR (immune checkpoint inhibitor[Title/Abstract])) OR (immune checkpoint inhibitors[Title/Abstract])) OR (ICIs[Title/Abstract])) OR (programmed cell death-1[Title/Abstract])) OR (programmed death-1[Title/Abstract])) OR (PD-1[Title/Abstract])) OR (programmed death-ligand 1[Title/Abstract])) OR (programmed death ligand 1[Title/Abstract])) OR (PD-L1[Title/Abstract])) OR (cytotoxic T-lymphocyte antigen-4[Title/Abstract])) OR (CTLA-4[Title/Abstract]))) AND ((hepatic arterial infusion chemotherapy[Title/Abstract]) OR (HAIC[Title/Abstract]))
Search Strategy for Embase	#1 'liver cell carcinoma'/exp OR 'liver cell carcinoma':ab,ti OR 'hepatocellular carcinoma':ab,ti OR 'liver cancer':ab,ti OR hcc:ab,ti<br>#2 'immunotherapy'/exp OR 'immunotherapy':ab,ti OR 'immune checkpoint inhibitor':ab,ti OR icis:ab,ti OR 'programmed death 1 receptor':ab,ti OR 'programmed death 1 ligand 1':ab,ti OR 'pd 1':ab,ti OR 'pd l1':ab,ti OR 'cytotoxic t lymphocyte antigen 4':ab,ti OR 'ctla 4':ab,ti<br>#3 'hepatic arterial infusion chemotherapy'/exp OR 'hepatic arterial infusion chemotherapy':ab,ti OR haic:ab,ti<br>#4 #1 AND #2 AND #3
Search Strategy for Cochrane Library	#1 MeSH descriptor: [Carcinoma, Hepatocellular] explode all trees<br>#2 ("hepatocellular carcinoma"):ti,ab,kw OR (HCC):ti,ab,kw OR ("liver cancer"):ti,ab,kw OR ("liver cell carcinoma"):ti,ab,kw<br>#3 #1 OR #2<br>#4 ("immunotherapy"):ti,ab,kw OR (immune checkpoint inhibitor):ti,ab,kw OR (immune checkpoint inhibitors):ti,ab,kw OR (ICIs):ti,ab,kw OR (programmed cell death-1):ti,ab,kw<br>#5 ("programmed death 1"):ti,ab,kw OR (PD-1):ti,ab,kw OR (programmed death-ligand 1):ti,ab,kw OR (programmed death ligand 1):ti,ab,kw OR (PD-L1):ti,ab,kw<br>#6 ("cytotoxic T-lymphocyte antigen 4"):ti,ab,kw OR ("cytotoxic T lymphocyte antigen-4"):ti,ab,kw OR ("cytotoxic T lymphocyte antigen 4"):ti,ab,kw OR (CTLA-4):ti,ab,kw

	#7 #4 OR #5 OR #6<br>#8 (hepatic arterial infusion chemotherapy):ti,ab,kw OR (HAIC):ti,ab,k<br>#9 #3 AND #7 AND #8

Supplementary Table 2. Assessment of Study Quality

First author (year)	Quality indicators from Newcastle-Ottawa Scale								
	**Selection (0-4)**				**Comparability (0-2)**	**Exposure/outcome (0-3)**			**Score (0-9)**
	**1**	**1**	**1**	**1**	**2**	**1**	**1**	**1**	**Total**
Zuo M (2025)	**1**	**1**	**1**	**1**	**2**	**1**	**1**	**1**	**9**
Lou Y (2025)	**1**	**1**	**1**	**1**	**1**	**1**	**1**	**1**	**8**
Zuo M (2024)	**1**	**1**	**1**	**1**	**2**	**1**	**1**	**1**	**9**
Wang X (2024)	**1**	**1**	**1**	**1**	**1**	**1**	**1**	**1**	**8**
Wang D (2024)	**1**	**1**	**1**	**1**	**1**	**1**	**1**	**1**	**8**
Li R (2024)	**1**	**1**	**1**	**1**	**2**	**1**	**1**	**1**	**9**
Guan R (2024)	**1**	**1**	**1**	**1**	**2**	**1**	**1**	**1**	**9**
Diao L (2024)	**1**	**1**	**1**	**1**	**1**	**1**	**1**	**1**	**8**
Chang X (2024)	**1**	**1**	**1**	**1**	**1**	**1**	**1**	**1**	**8**
Fu Y (2023)	**1**	**1**	**1**	**1**	**2**	**1**	**1**	**1**	**9**
Mei J (2021)	**1**	**1**	**1**	**1**	**1**	**1**	**0**	**1**	**7**
Chen S (2021)	**1**	**1**	**1**	**1**	**1**	**1**	**0**	**1**	**7**

NEWCASTLE - OTTAWA QUALITY ASSESSMENT SCALE

COHORT STUDIES

Note: A study can be awarded a maximum of one star for each numbered item within the Selection and Outcome categories. A maximum of two stars can be given for Comparability
